# The heat shock response and humoral immune response are mutually antagonistic in honey bees

**DOI:** 10.1038/s41598-017-09159-4

**Published:** 2017-08-18

**Authors:** Mia McKinstry, Charlie Chung, Henry Truong, Brittany A. Johnston, Jonathan W. Snow

**Affiliations:** 1 Biology Department, Barnard College, New York, NY 10027 USA; 2 Natural Sciences Department, LaGuardia Community College-CUNY, Long Island City, NY 11101 USA; 3 Biology Department, The City College of New York-CUNY, New York, NY 10031 USA

## Abstract

The honey bee is of paramount importance to humans in both agricultural and ecological settings. Honey bee colonies have suffered from increased attrition in recent years, stemming from complex interacting stresses. Defining common cellular stress responses elicited by these stressors represents a key step in understanding potential synergies. The proteostasis network is a highly conserved network of cellular stress responses involved in maintaining the homeostasis of protein production and function. Here, we have characterized the Heat Shock Response (HSR), one branch of this network, and found that its core components are conserved. In addition, exposing bees to elevated temperatures normally encountered by honey bees during typical activities results in robust HSR induction with increased expression of specific heat shock proteins that was variable across tissues. Surprisingly, we found that heat shock represses multiple immune genes in the abdomen and additionally showed that wounding the cuticle of the abdomen results in decreased expression of multiple HSR genes in proximal and distal tissues. This mutually antagonistic relationship between the HSR and immune activation is unique among invertebrates studied to date and may promote understanding of potential synergistic effects of disparate stresses in this critical pollinator and social insects more broadly.

## Introduction

Honey bee colonies in the United States have suffered from a higher than usual rate of mortality in the last few years with a complex set of interacting stresses playing a key role. Some stresses thought to be involved include nutritional stress due to loss of appropriate forage, chemical poisoning from pesticides, changes to normal living conditions brought about through large-scale beekeeping practices, and infection by pathogenic microbes^1^. In seeking to understand how stresses might synergize to impact honey bee health, efforts have been undertaken to more completely define common cellular processes and cell stress pathways that are impacted by multiple stressors.

One such process is proteostasis, which refers to the homeostasis of protein synthesis, folding, function, and degradation both within a cell and in an organism as a whole^2^. A number of normal and pathologic conditions can lead to disruption of proteostasis. This creates a build-up of unfolded proteins in the cell, triggering a suite of responses designed to limit resulting damage and return the cell to homeostasis^2^. Within individual cells, proteostasis is maintained by the cellular stress responses of the proteostatic network. These responses include the Heat Shock Response (HSR)^3, 4^ responding to proteostatic disruption in the cytoplasm, the endoplasmic reticulum Unfolded Protein Response (UPR^ER^) responding to proteostatic perturbation in the endoplasmic reticulum^5^, and the mitochondrial Unfolded Protein Response (UPR^mt^) responding to proteostatic perturbation in the mitochondria^6^. As conditions leading to unfolded proteins can be caused by perturbation of multiple cellular processes and pathways, the proteostastic network provides an optimal hub for sensing and responding to cellular stresses of myriad origin.

The HSR has been well characterized in the invertebrate models *Drosophila melanogaster* and *Caenorhabditis elegans* ^2, 4^. Due to their particular lifestyle, honey bees are exposed to significant routine proteostatic stressors suggesting that the HSR might have unique properties in these insects. Colony-level homeostatic regulation of hive temperature is a key feature of honey bee colony function^7–11^. Hive temperature is carefully maintained between 32° and 35 °C during normal conditions in colonies in temperate regions. This narrow temperature range is important for brood development and normal colony function^12–15^. Complex individual behaviors, including endothermic shivering to increase temperature, are utilized by individual bees to maintain this relatively constant temperature^10^ (and references therein). In maintaining this narrow range of hive temperature and in performing other specialized tasks, the temperature of individual bees can increase significantly above steady-state to levels that are dangerous to other organisms. For example, the temperature of individual forager bees can reach up to 49 °C in flight^16^. Honey bees appear highly resistant to heat-shock^17–19^, yet experimental evidence to date is contradictory with regards to the presence of a robust heat-shock response in honey bees^17, 18, 20^ and our understanding of the specific molecular components involved in performance and regulation of this response is incomplete.

Considering the many known microbial threats to the honey bee, including bacterial, viral, fungal, and parasitic^21^, we were particularly interested in how the HSR might interact with honey bee immune activation. In studies of invertebrates to date, evidence supports the existance of a positive reciprocal relationship between the HSR and immune activation^22^. Immune competence, resistance to infection, and levels of immune mediators all increase with temperature and heat shock. In return, infection enhances temperature stress tolerance and expression of HSR target genes. However, it is likely that the individual response to interacting stresses in solitary insects, in which these prior studies were performed, and eusocial insects, such as the honey bee, are divergent. One of the key features leading to the ‘resilience’ of eusocial organisms appears to be the ability to sacrifice non-reproductive individuals for the benefit of the colony^23^. Thus, individual worker honey bees may have different stress response characteristics in the context of the colony, especially when confronted simultaneously by multiple stresses.

Here, we demonstrate that the core components and function of the HSR pathway are conserved in the honey bee. In addition, our results demonstrate that heat shock decreases the expression of specific immune effectors, namely the antimicrobial peptide genes *Hymenoptaecin*, *Defensin 1*, and *Abaecin*. Inversely, wounding of the abdomen results in decreased expression of multiple HSR target genes in proximal and distal tissues, revealing a mutually antagonistic relationship between the HSR and immune activation in honey bees. This relationship is unique among invertebrates studied to date and may have important consequences for understanding potential synergistic effects of disparate stresses in this critical pollinator and social insects more broadly.

## Results

### The HSR pathway is conserved in honey bees

We first examined the honey bee genome and identified the gene encoding the canonical Heat Shock Factor (HSF), which is the transcriptional regulator of the HSR, and is found in most eukaryotes. Similarly to *D. melanogaster* ^24^, honey bees possess a single gene encoding HSF. Recent work has identified a core set of HSF-dependent genes in the yeast *Saccharomyces cerevisiae* ^25^ and in mammals^25, 26^. We used these to identify putative homologs (Table 1) and found that *A. melliferra* possess apparent homologs for all of the proposed transcriptional targets of the pathway except *Dedd2*. However, some differences were evident. Honey bees possess two *Hsp90* genes as reported before, *Hsp90* and *Hsp83l* ^27^. In addition, we found that the honey bee possesses two H*sp*70 genes that correspond to three H*sp*70 genes (H*spa1a*, *Hsp70Ab/Hspa1*, and *Hspa8*) described as HSF-core targets in mammals. We also used information in *D. melanogaster* and *Homo sapiens* to identify other genes encoding chaperone proteins of the HSP70, HSP90, DNAJ-containing, and alpha-crystallin/sHSP families in the *A. mellifera genome*. The honey bee genome possesses three additional genes encoding HSP70 in addition to the ones shown in Table 1, including *Hsc70-3*, *Hsc70-5*, and *Hyuop1-like* (all genes encoding HSP70 proteins are listed in Suppl. Table 2). There are two additional genes encoding HSP90 proteins in the honey bee genome in addition to the ones shown in Table 1 (all genes encoding HSP90 proteins are listed in Suppl. Table 3). In addition to the ones shown in Table 1, the honey bee genome also contains 25 further genes encoding proteins containing DNAJ motifs (all genes encoding DNAJ-containing proteins are listed in Suppl. Table 4). There are thirteen genes encoding proteins containing the alpha-crystallin domain characteristic of small heat shock proteins in the honey bee genome (all genes encoding alpha-crystallin domain-containing proteins are listed in Suppl. Table 5). These proteins play an important ‘first line’ role in maintaining proteostasis^28^. In addition to *Hsp10* (shown in Table 1), the honey bee genome has the larger subunit of the mitochondrial, Group I Chaperonin, *Hsp60*. In eukaryotic organisms, the proteins of the Group II Chaperonin, the TCP-1 Ring Complex **(**TRiC) are also involved in folding^29^, and the honey bee possesses genes encoding all eight expected subunits (all genes encoding chaperonin proteins are listed in Suppl. Table 6).

**Table 1:** *Apis mellifera* homologs of core HSF-dependent HSR genes identified in other species.

*D. melanogaster* gene (*H. sapiens* name)	*A. mellifera* homolog (name)	Gene ID	proposed function
*Heat shock factor* (*Hsf1*)	XP_395321.3 (*Hsf*)	411854	HSF
*Hsc70-4*, *Hsp70Ab* (*Hspa1a*, *Hspa8*, *Hspa1*)	NP_001153544.1, NP_001153522.1, (*Hsc70-4*, *Hsp70Ab*)	410620, 409418	HSP70
*Hsp90* (*Hsp90ab1*), *Hsp83l*	NP_001153536.1, XP_006571335.1(*Hsp90, Hsp83l*)	408928, 411700	HSP90
*Hsp70Cb* (*Hsph1*)	XP_006561225.1(*Hsp70Cb*)	408706	HSP110
*CG5001*, *Dnaj1*(*Dnajb1*)	XP_006562214.1(*Dnaj1*)	411071	HSP40
*DnaJ-like-2*(*Dnaja1*)	XP_006566003.1(*Dnaja1*)	724368	HSP40
*CG11267* (*Hspe1*)	XP_624910.1(Hsp10)	552531	mtHSP10
(*Dedd2*)	not found	NA	NA

### Heat-shock induces a classic HSR in honey bees

**Figure 1:**
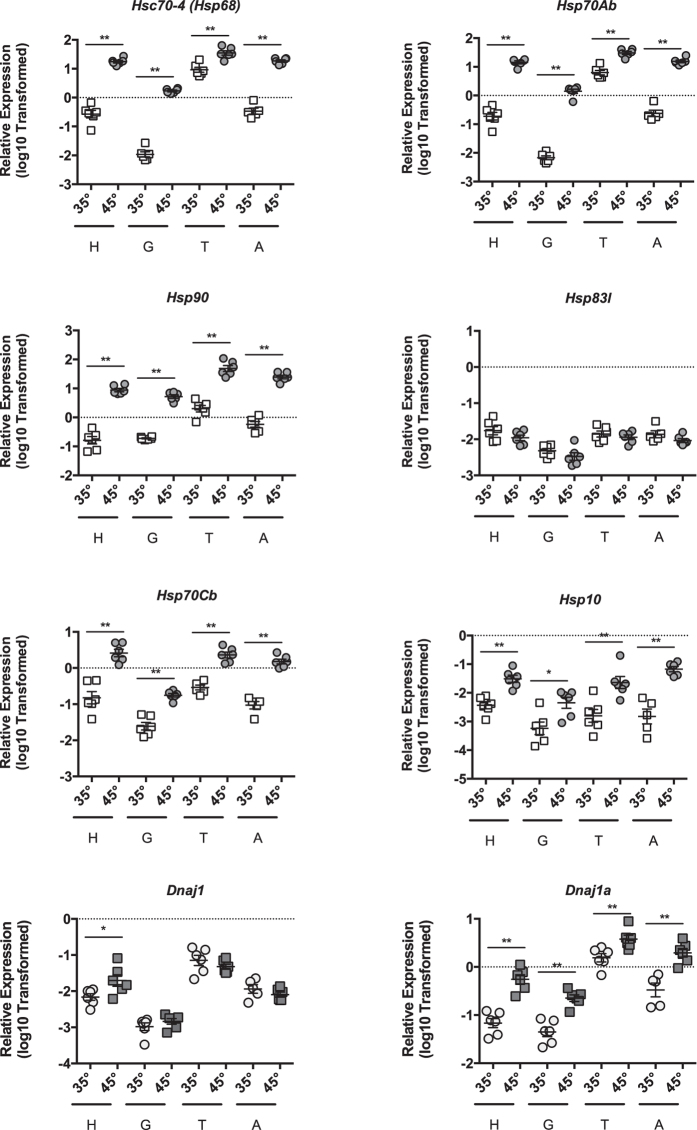
Core HSR target genes are induced during Heat-Shock. Transcript levels of putative core HSR target genes *Hsc70-4*, *Hsp70Ab*, *Hsp90*, *Hsp83l*, *Hsp70Cb*, *Hsp10*, *Dnaj1*, and *Dnaja1* relative to *β-actin* in head tissue (H, predominantly brain and sensory organ tissue), midgut (G), thorax tissue (T, predominantly flight muscle), and abdominal wall tissue (A, predominantly fat body) from adult bees captured at the landing board and maintained for four hours in cages at either 35° or 45 °C. Symbols represent expression values of the genes of interest calculated using the ΔΔC_T_ method for individual bees. Mean ± SEM is also shown. Statistical significance was assessed using unpaired t-tests with Welch’s and is noted as *p < 0.05, and **p < 0.01.

We examined heat-shock dependent induction of putative heat-shock genes in head tissue (predominantly brain and sensory organ tissue, n = 6), midgut (n = 6), thorax tissue (predominantly flight muscle, n = 6), and abdominal wall tissue (predominantly fat body, n = 6 for 35° and n = 5 for 45°) at 35° and 45 °C for 4 hours. Relative to *β-actin*, we observed robust induction of the homologs of the core HSF target genes, *Hsc70-4*, *Hsp70Ab*, *Hsp90*, *Hsp70Cb*, *Dnaja1*, and *Hspe1* in all tissues examined (Fig. 1). However, we did not observe induction of the other Hsp90 family member gene, *Hsp83l* (Fig. 1). Additionally, *DnaJ protein homolog 1-like* (*Dnaj1*) was only induced in the head tissue (Fig. 1). *β-actin* levels were similar irrespective of temperature as assessed by Ct values (Suppl Figure 2). As expected, the transcriptional regulator, *Hsf*, was not induced after heat shock (Suppl Figure 2). In addition, although we observed a robust induction of these heat-shock target genes in all four tissues, the magnitude of induction differed between tissues.

### Targets of the broader proteostasis network are induced in response to heat-shock

**Figure 2:**
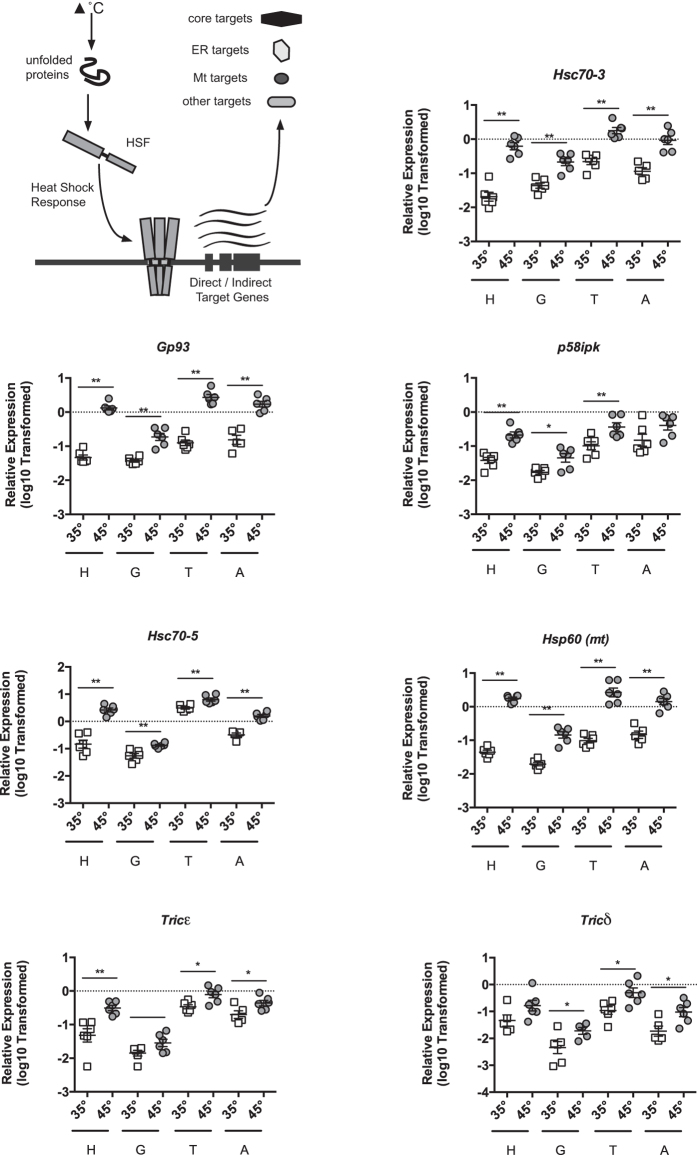
Select UPR^ER^, UPR^mt^, and broader cytoplamsic target genes are induced during heat shock. Graphical schematic of broader targets examined (upper left). Transcript levels of UPR^ER^ targets genes *Hsc70-3*, *Gp93*, *p58ipk*, the UPR^mt^ targets genes *Hsc70-5*, *Hsp60*, and TRiC genes representing the *Tricε* and *Tricδ* subunits relative to relative to *β-actin* in head tissue (H, predominantly brain and sensory organ tissue), midgut (G), thorax tissue (T, predominantly flight muscle), and abdominal wall (A, predominantly fat body) from adult bees captured at the landing board and maintained for four hours in cages at either 35° or 45 °C. Symbols represent expression values of the genes of interest calculated using the ΔΔC_T_ method for individual bees. Mean ± SEM is also shown. Statistical significance was assessed using unpaired t-tests with Welch’s and is noted as *p < 0.05, and **p < 0.01.

We also explored potential targets from the broader proteostasis network. *Hsc70-3*, which is a characterized target of the Unfolded Protein Response (UPR), was upregulated by heat shock (Fig. 2). Thus, we wondered if other UPR target genes would be induced as well. Using previously identified honey bee UPR target genes^30^, we found that a subset of these, *Gp93* and *p58ipk*, (induction in the abdomen displayed a strong trend towards significance, p = 0.082), were triggered by heat shock in most honey bee tissues (Fig. 2). We also found that a greater number of UPR target genes were upregulated in only one or two tissues (Suppl Figure 3). The ER-localized GRP170 homolog, *Hyuop1*-*like*, was not affected by heat shock (Suppl Figure 3). We wished to determine whether heat shock induction of UPR^ER^ target genes was occurring in conjunction with UPR pathway activation or in a potentially UPR-independent manner. IRE1, a transmembrane receptor involved in one of three UPR^ER^ pathways, is usually bound to the ER chaperone HSC70-3 and maintained in a monomeric, inactive form. Upon increase of unfolded proteins in the lumen of the ER, IRE1 is activated by loss of HSC70-3 binding. This leads to IRE1 multimerization and ultimately to activation of its endonuclease domain, which performs non-canonical splicing of the mRNA encoding the bZIP transcription factor XBP1. In its unspliced form, the *Xbp1* mRNA (*Xbp1u*) encodes a truncated protein (XBP1u) with low transactivation activity. Splicing removes a short sequence containing an in-frame stop codon, leading to the translation of the new transcript (*Xbp1s*), which encodes a longer form of XBP1 (XBP1s) with enhanced transactivation activity, leading to target gene induction. We previously characterized the IRE1 pathway in honey bees and developed a method for measuring its activation. Examination of *Xbp1* mRNA splicing in the midguts of bees exposed to heat-shock revealed that neither bees caged at 35 °C nor those incubated at 45 °C had detectable *Xbp1* mRNA splicing (Suppl. Figure 3B) after 4 hours, at a time when UPR targets are transcriptionally upregulated.

In addition, as the mitochondrially localized *Hsp10* was upregulated by heat shock (Fig. 1F), we wished to determine whether other UPR^mt^ targets were activated during the heat shock response in honey bees. Heat stress affects protein-folding in the mitochondria and activates the UPR^mt^ ^31^. Examining other putative honey bee UPR^mt^ target genes, we found that two targets, *Hsc70-5* and *Hsp60*, were induced by this treatment (Fig. 2). Two other UPR^mt^ targets, the HSP90 family member *Trap1* and the DNAJ-containing protein *Tim14*, were not activated during the heat shock response in honey bees (Suppl. Figure 4). While the subunits of the TRiC complex are also involved in folding, they are not upregulated by the HSR upon cytoplasmic proteostatic stress in other organisms^32^. However, we found that the *T*-*complex chaperonin epsilon* (T*ricε*) (induction in the abdomen displayed a strong trend towards significance, p = 0.056)) and *T*-*complex chaperonin* delta (T*ricδ*) (induction in the abdomen displayed a strong trend towards significance, p = 0.088), subunits were induced in most tissues when bees were exposed to 45 °C (Fig. 2). By contrast, the *T*-*complex chaperonin eta* (*Tricη*) and T-*complex chaperonin thelta* (*Tricθ*) subunits were not induced (Suppl Figure 4). In addition to those already tested, we assessed the effect of heat shock on 4 other genes encoding DNAJ proteins based on the localization and known client binding characteristics of their human homologs as described in Kampinga *et al*.^33^, testing all the cytoplasmically localized DNAJ-containing proteins with promiscuous or wide client binding function, *DnajA3*, *DnajB11*, *DnajB12*, *DnajB6* (Suppl Figure 4).

### Proteasome-inhibitors induce HSR in honey bees

**Figure 3:**
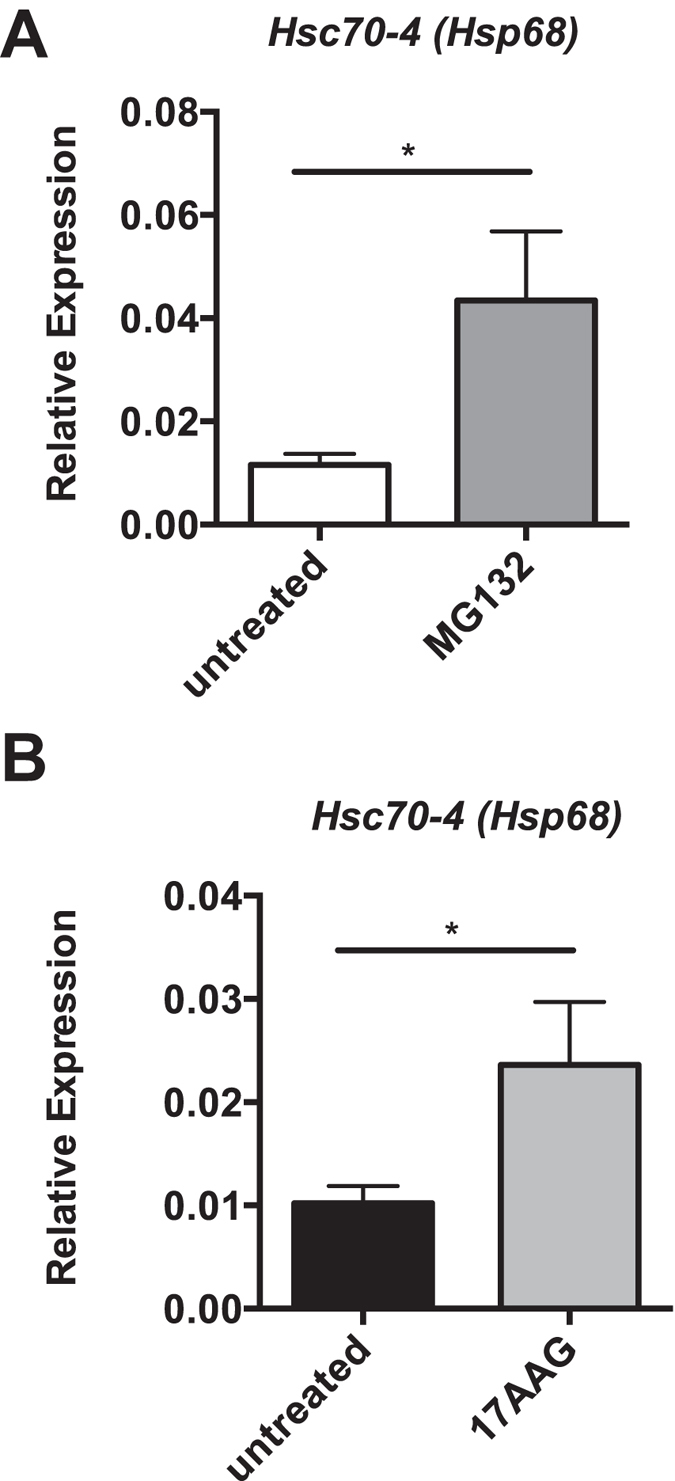
Pharmacological induction of the HSR. Individual levels of cytoplasmic chaperone *Hsc70-4* transcripts relative to *β-actin* in midgut tissue after MG132 (**A**) or 17AAG (**B**) treatment relative to untreated bees. Bar and error bars represent the Mean ± SEM for expression values of the genes of interest calculated using the ΔΔC_T_ method. Statistical significance was assessed using unpaired t-tests with Welch’s and is noted as *p < 0.05, and **p < 0.01.

Inhibition of the proteasome causes a build-up of misfolded proteins in the cytoplasmic compartment and is commonly used to trigger HSR in a variety of model organisms^24^. Therefore, we examined heat shock-independent induction of cytoplasmic chaperones in midguts from bees treated with the proteosome inhibitor MG132, which has been used in other invertebrates^34, 35^, for 24 hours. Relative to *β-actin*, we observed robust induction of the cytoplasmic Hsp70 family member, *Hsc70-4* (*Hsp68*), when bees were exposed to 500 µM MG132 (n = 9) compared to untreated bees (n = 6) (Fig. 3A).

### Honey bees HSR is regulated by HSP90

In other species, HSP90 is the major negative regulator of HSF function. In these organisms, inhibitors which prevent its binding to HSF can cause a HSR in the absence of misfolded proteins. Therefore, we examined heat shock-independent induction of cytoplasmic chaperones in midguts from bees treated with the HSP90 inhibitor, 17-(Allylamino)-17-demethoxygeldanamycin (17-AAG), which has been used in *Drosophila* ^36^, for 24 hours. Relative to *β-actin*, *Hsc70-4* (*Hsp68*) was induced when bees were exposed to 250 µM 17-AAG (n = 9) compared to untreated bees (n = 6) (Fig. 3B).

### Heat shock represses expression of immune effectors

**Figure 4:**
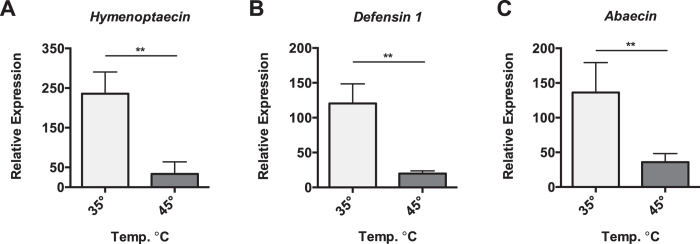
Select AMP gene repression during Heat-Shock. Transcript levels of *Hymenoptaecin*, *Defensin 1*, and *Abaecin* relative to *β-actin* in abdominal wall (predominantly fat body) from bees maintained for four hours in cages at either 35° or 45 °C. Bar and error bars represent the Mean ± SEM for expression values of the genes of interest calculated using the ΔΔC_T_ method. Statistical significance was assessed using unpaired t-tests with Welch’s and is noted as *p < 0.05, and **p < 0.01.

In most invertebrates examined to date, heat shock induces the expression of immune genes responsible for defending against microbial infection, likely due to hormetic cross-activation of these stress responses. We were interested in determining whether this relationship between the HSR activation and immune gene expression extended to honey bees. Surprisingly, we observed substantial repression of multiple AMP genes, *Hymenoptaecin*, *Defensin 1*, and *Abaecin*, in the abdominal tissue (containing the fat body) when bees were exposed to 45 °C for 4 hours compared to bees maintained at 35 °C (Fig. 4A–C).

### Septic infection reveals a reciprocal relationship between wounding and heat shock targets

**Figure 5:**
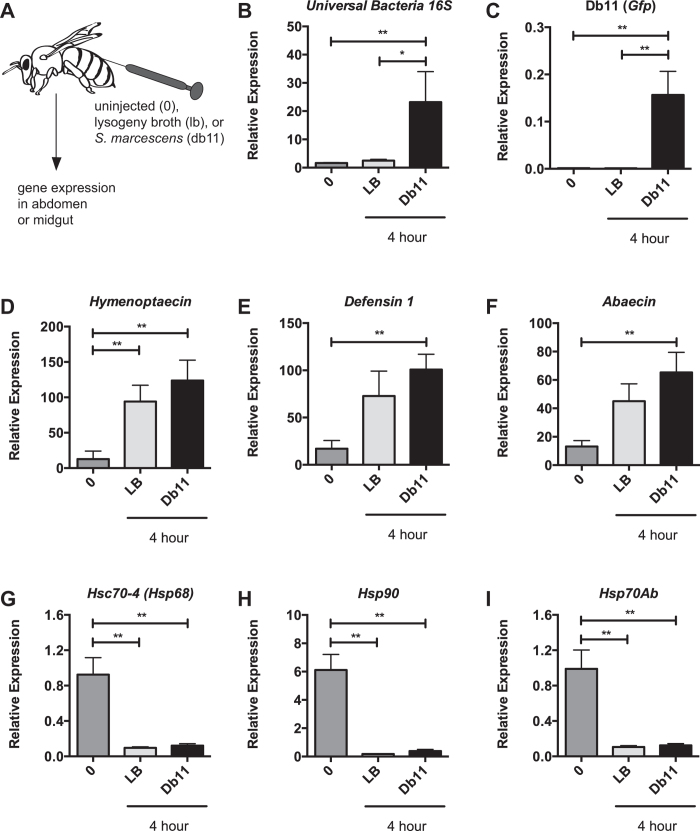
Infection and wounding represses select HSR target genes at the site of injury. Graphical schematic of infection protocol (**A**). Levels of bacteria (**B**) or *Gfp1* + *S. marcescens* (Db11) (**C**) relative to *β-actin* in abdominal wall (predominantly fat body) in uninjected bees (0) or those injected with either lysogeny broth (LB) or *S. marcescens* (Db11) four hours after injection. Transcript levels of *Hymenoptaecin* (**D**), *Defensin 1* (**E**), and *Abaecin* (**F**) or *Hsc70-4* (**G**), *Hsp70Ab* (**H**) and *Hsp90* (**I**) relative to *β-actin* in abdominal wall (predominantly fat body) from these same bees. Bar and error bars represent the Mean ± SEM for expression values of the genes of interest calculated using the ΔΔC_T_ method. Statistical significance was assessed using one way ANOVA with Tukey’s multiple comparison test and is noted as *p < 0.05, and **p < 0.01.

We were interested in determining whether there was a reciprocal relationship between the HSR and microbial infection and established a septic infection model using the Gram-negative bacteria, *S. marcescens* (Fig. 5A). We observed increased levels of total bacteria (using Universal Bacteria *16 S* primers) and *Gfp* (expressed in the Db11 *S. marcescens* clone) in the fat body of bees receiving *S. marcescens* (n = 4) relative to uninjected bees (n = 4) and those injected with Lysogeny Broth (LB) alone (n = 4) (Fig. 5B and C). After injection of Gram-negative bacteria (*S. marcescens*) or media alone, we observed robust activation of AMPs in the fat body compared to uninjected bees at 4 hours post-injection (Fig. 5D–F). We also observed a significant reduction in the expression of genes encoding the two HSP70 proteins, *Hsc70-4* and *Hsc70Ab*, and *Hsp90* (Fig. 5G–I). These results suggest that wounding is sufficient for immune activation and HSR repression regardless of the presence of microbes.

To determine if wounding was sufficient for altering the expression of proteostasis genes, we used a wounding model in which we made a sterile injury in the cuticle of the honey bee abdomen. When we performed this challenge, we again observed robust activation of the AMPs *Abaecin*, *Hymenoptaecin*, and *Defensin 1* in the fat body of wounded (n = 8) compared to unwounded (n = 8) bees (Suppl. Figure 5A and C). We also observed a significant reduction in the genes encoding the two HSP70 proteins, *Hsc70-4* and *Hsp70Ab* (Suppl. Figure 5D and E), and *Hsp90* (Suppl Figure 5F).

**Figure 6:**
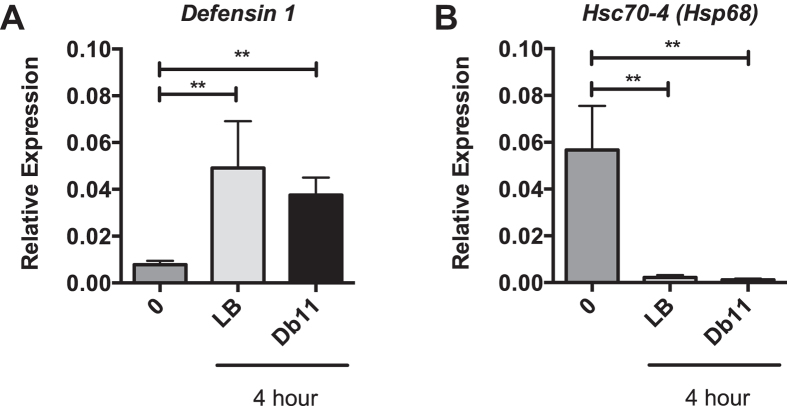
Infection and wounding represses select HSR target genes at the site of injury. Transcript levels of *Defensin 1* (**A**), *and Hsc70-4* (**B**) relative to *β-actin* in midgut tissue in uninjected bees (0) or those injected with either lysogeny broth (LB) or *S. marcescens* (Db11) four hour after injection. Bar and error bars represent the Mean ± SEM for expression values of the genes of interest calculated using the ΔΔC_T_ method. Statistical significance was assessed using one way ANOVA with Tukey’s multiple comparison test and is noted as *p < 0.05, and **p < 0.01.

Spreading of immune and proteostasis signals beyond the site of the insult have been reported. Examination of gene expression in the midgut tissue of bees from the septic infection model revealed robust activation of the AMP *Defensin 1* compared to uninjected bees (Fig. 6A) and a significant reduction in the genes encoding the HSP70 protein, *Hsc70-4* (Fig. 6B). Alterations in gene expression distal from the site of wounding implies that some signal is allowing for spread of both immune activation and HSR repression and that this signal does not require the presence of bacteria.

## Discussion

Our understanding of the molecular architecture and regulation of the HSR in honey bees is incomplete^17, 18, 20^. In other organisms, activation of the HSR pathway leads to the activation of a family of leucine-zipper containing transcription factors, HSF, which participate in a medium-term response designed to increase protein production and folding capacity through transcriptional upregulation of proteins involved in these processes^3, 4^. A number of recent studies have also suggested that within the myriad transcriptional changes induced by heat-shock there is a core set of HSF-dependent genes shared in the HSR from yeast^25^ to mammals^25, 26^, although some differences in this core group were found in a similar study in *C. elegans* ^37^. Our findings demonstrate that temperatures encountered by honey bees during normal activities result in robust HSR induction and increased expression of the majority of the homologs of the core HSF-dependent found in other systems. Ultimately however, genetic approaches will be necessary to definitively determine HSF1-dependence of these genes in the honey bee HSR.

At least two other pathways contribute to the maintenance of proteostasis in cells, the UPR^ER^, which responds to proteostatic stress in the endoplasmic reticlulum^5^ and the UPR^mt^, which responds to proteostatic stress in mitochondria^6^. We have previously characterized the honey bee UPR^[ER 30^ and find here that select genes involved in the unfolded protein response in the ER are also triggered by the HSR. We also find that the IRE1 pathway does not appear to be activated by heat shock as assessed by *Xbp1* splicing. *Xbp1* is upregulated by heat shock in some tissues. However, some evidence suggests that unspliced XBP1 represses target genes and *Xbp1* levels do not correlate with increased UPR^ER^ target gene expression in our experiments. This suggests that the heat shock response is directly activating these targets via HSF, some unknown intermediary, or one of the other UPR pathways, such as the activating transcription factor 6 (ATF6) or double-stranded RNA-activated protein kinase (PKR)–like ER kinase (PERK) pathway. While the UPR^mt^ has not yet been characterized in this species, we observe activation of honey bee homologs of select genes that are part of this response in other species^38^. In addition, it is likely that HSR activation shown here impacts other processes involved in proteostasis, such as the ubiquitin proteasome system (UPS)^39^, which has been characterized in honey bees^40, 41^. Heat shock likely impacts protein folding in all compartments, however there is accumulating evidence that in some contexts, proteotoxic insults isolated to one cellular compartment communicate this insult to other compartments resulting in cell-wide responses^42, 43^. Further work will be necessary to fully understand the biological importance and mechanistic underpinnings of proteostasis network crosstalk in this species and other insects.

The honey bee immune system is well characterized^44^, possessing the machinery for both the Toll and IMD pathways involved in Pathogen-Associated-Molecular-Pattern (PAMP) recognition^45^. Functional data suggests that septic infection by Gram-negative or Gram-positive bacteria in honey bees results in activation of a systemic immune response in a manner that is not pathogen-specific^46^. Our data here also suggests a PAMP-independent activation of the immune system in honey bees as we find that the AMPs *Hymenoptaecin*, *Defensin 1*, and *Abaecin* are induced by sterile wounding alone. Immune activation in invertebrates can be triggered by two mechanisms other than these PAMP-dependent pathways. First, sterile mechanical wounding of the epidermis in multiple invertebrate species, including *C. elegans* ^47^, *D. melanogaster* ^48^, *Spodoptera frugiperda* ^49^, *Bombus terrestris* ^50^, and honey bees^51, 52^, can elicit an immune response that includes AMP production, perhaps as prophylactic protection against anticipated infection after wounding. Second, ‘surveillance immunity’ is triggered through the sensing of perturbations in normal cellular processes^53, 54^. In particular, disruption of proteostasis often triggers this response in invertebrates^55^. For example, translation inhibition often occurs during microbial infection in worms^56–58^ and flies^59^, resulting in immune activation. It is likely that mechanical damage, cellular disruption, or both is leading to immune activation in the epidermis and fat body here as well. While we only examined the systemic humoral response in the form of AMP expression in this study, systemic immunity in insects is made of both cellular and humoral elements^45^ and future studies might explore the effect of heat shock on other aspects of the immune response.

Prior to this report, evidence has suggested a positive reciprocal relationship between the HSR and immune activation in invertebrates^22^. Immune competence, resistance to infection, and levels of immune mediators all increase with temperature and heat shock. For example, increased temperature makes *Galleria mellonella* more resistant to infection with fungi^60–62^ and bacteria^63^. Heat shock was also shown to result in induced expression of humoral immune genes^64, 65^. Temperature also positively impacted the expression of immune response genes in the alfalfa leafcutting bee^66^. In *C. elegans*, heat increases the immune response^67^ and HSF is also important for immune mediated resistance to infection^68, 69^. In *Drosophila*, the p38 pathway mediates defense to bacterial and fungal infections in part through a HSF-dependent mechanism^70^. Evidence also suggests that activation of the HSR inhibits virus infection^71^. Reciprocally, in other invertebrate models, infection appears to enhance temperature stress tolerance, stress responses, and expression of stress genes, including HSR target genes. Bacterial infection induced *Hsp90* expression in the greater wax moth^72^ and *Hsp70* expression in *Musca domestica* ^73^. Hormesis, the beneficial effects of a treatment that are harmful at a higher intensity^74^, may be the underlying mechanism in these other invertebrates. Sublethal exposure to heat stress or immune stress may induces a cellular or physiological response that results in cross-resistance to the other seemingly unrelated stress.

Our results in the honey bee are consistent with the idea of trade-offs from ecological immunology^75–77^. One tenet of this field is that processes that compete for similar resources, such as energy, can come into conflict at the physiological level resulting in unbalanced emphasis on one or the other system^78^. The immune system is costly and its activation is often used as one of the competing processes in studies of trade-offs. Cellular mechanisms to maintain proteostasis are also energetically costly^79^. Thus, energetic trade-offs at the individual level would be expected to allocate energy resources in a manner that leads to a reduction in either immunocompetence or proteostasis. One reason for the differences observed in this study could be that individual worker honey bees have different stress response requirements as a eusocial species. Specifically, in the context of the colony, limited resources are allocated to the individuals most likely to benefit the hive. Originally described in the context of aging and senescence in social insects, the intergenerational transfer theory of aging proposes that allocation of group resources to the non–reproductive individuals, such as workers, will be governed by the amount of resource transfers each individual is likely to provide to the group^80, 81^. Stressed individuals are less productive and will deliver fewer resource transfers to the group and thus may not be prioritized for receiving limited group resources. For example, the stress of immune activation has been observed to alter learning^82^ and both individual^83^ and social^84, 85^ behavior in honey bees, all of which could impact the ability of individual bees to contribute to the colony. In fact, stresses encountered by individuals of this species often alter physiology and behavior to increase participation in the most risky colony tasks, such as foraging^86^ or robbing^87^. For example, a wide array of stresses, including nutritional deficiencies^88, 89^, infection^83, 90–95^, and immune stimulation^96, 97^ will lead to precocious foraging and forager physiological features in younger nurse bees^98^. Early foragers are less successful and die earlier, which results in an increased rate of forager recruitment. While, the ‘resilience’ of eusocial organisms appears to rely on the ability to sacrifice non-reproductive individuals for the benefit of the colony^23^, even in the group context the supply of individuals is limited and sustained loss can have dramatic impacts on colony fitness^99^.

All metazoan species possess means for coordinating responses between tissues and organs, especially in cases of stress^100^. Spreading of immune^101^ and proteostasis^2, 102^ signals beyond the site of the insult have been reported in invertebrates. Our discovery that alterations in immune and proteostasis gene expression are occurring in the midgut, which is distal from the site of wounding, implies that some signal is allowing spread of these signals in honey bees as well. For both signal types, communication appears to involve both neural networks^103, 104^ and soluble hormone signaling^105^. Communication between the nervous system and distal tissues is bidirectional. For example, in *C. elegans*, thermosensory neurons contribute to the regulation of HSR in other tissues^106^ and temperature sensing in disparate tissues can affect behavioral responses to temperature^107^. More work to understand how the thermosensory system functions in bees at the individual and colony level under normal conditions is warranted. Honey bees participate in activities to maintain a relatively constant hive temperature^10^ (and references therein) that is critically important for normal brood development and normal colony function. In particular, development outside of the optimal temperature range results in brain abnormalities, cognitive defects, and reduced survival^12–15^. In light of the influence of proteostasis on behavioral responses in the roundworm, it is interesting to speculate on how wounding or immune activation in honey bees could impact thermoregulatory behavior via modulation of the proteostasis pathways. For example, *Varroa destructor* is an ectoparasitic mite that attacks the honey bee^108^. Its mode of feeding involves puncturing the cuticle to suck hemolymph. A recent report found that *Varroa* infection and artificial wounding elicited similar transcriptional responses^109^. In addition, infestation of colonies by V. *destructor* interferes with proper thermoregulation in honey bee winter clusters, specifically leading to higher thermal fluctuations^110^. Thus, the role of wounding and the HSR in this phenomenon should be explored.

In the characterization of the honey bee HSR described here, we found that the core components of the pathway are conserved and that temperatures encountered by honey bees during normal activities resulted in robust HSR induction and the increased expression of specific heat shock proteins in a manner that was variable across tissues. In addition, we show that heat shock represses the expression of select immune effectors, the antimicrobial peptide genes, *Hymenoptaecin*, *Defensin 1*, and *Abaecin*. Finally, we show that wounding the abdomen results in decreased expression of multiple HSR target genes in proximal and distal tissues, revealing a reciprocally antagonistic relationship between the HSR pathway and immune activation in honey bees, which is distinctive among invertebrates studied to date. These findings may offer new insight into the potential synergistic effects of disparate stresses in this critical pollinator and social insects more broadly.

## Materials and Methods

### Honey bee Tissue Collection

Honey bees were collected from the landing board of outbred colonies in New York, New York consisting of a typical mix of *Apis mellifera* subspecies found in North America, at different times during the months of April-October. Only visibly healthy bees were collected and all source colonies were visually inspected for symptoms of common bacterial, fungal, and viral diseases of honey bees. After cold anesthesia, bees were dissected and we recovered four tissues for analysis; head tissue (predominantly brain and sensory organ tissue including antennae), midgut, thorax tissue (predominantly flight muscle), and abdominal wall (predominantly fat body). Tissues were set aside for gene expression analysis by storing in RNAlater (Invitrogen, San Diego, CA).

### Ortholog screening of the honey bee genome

HSR pathway gene candidates from *D. melanogaster* were used to find orthologs in the honey bee genome using the BLAST family of search functions (www.ncbi.nlm.nih.gov) as described previously^30^. The KEGG (Kyoto Encyclopedia of Genes and Genomes) database was also used as a guide for comparing pathways between species^111^. For proteins of interest, we used SignalP 4.1 to predict the presence of a signal sequence, TargetP 1.1 to predict secretory or mitochondrial localization, and Prosite to predict the presence of an ER-retention signal.

### Temperature and Chemical Treatments

For all caged experiments, honey bees were selected as above and kept in 177.4 mL (6 oz.) Square-bottomed *Drosophila* Stock Bottles (VWR) plugged with modified foam tube plugs (Jaece Industries). Bees were maintained in incubators at 35 °C (unless otherwise stated) in the presence of PseudoQueen (Contech, Victoria, British Columbia, Canada) as a source of Queen Mandibular Phermone (QMP) and used as per manufacturer’s instructions. For heat shock, bees were maintained for four hours in cages at 35° or 45 °C. Bees were fed 33% sucrose via a modified 1.5 ml screw-cap tube, with or without the following chemicals at the indicated doses: 500 µM MG132^34, 35^ (Sigma), 250 µM 17-(Allylamino)-17-demethoxygeldanamycin (17-AAG) (Calbiochem)^36^ for 24 hours. When DMSO was used as the chemical solute, equivalent amounts of DMSO were added to the food of the control group.

### Bacterial Infections

For bacterial infection, *Serratia marcescens* (strain Db11^112^) from frozen stocks were plated on Lysogeny Broth (LB) Agarose with Ampicillin and Streptomycin, a colony was selected to be grown in LB without antibiotics overnight, and the resulting culture was diluted in fresh LB without antibiotics. Bacteria was then grown exponentially at 37 **°**C to OD600 = 1. Db11 cultures or LB alone were then diluted 1:10 in Ringer’s solution. For injections, bees were cold anesthetized, immobilized, and 1 μl was injected between the 2^nd^ and 3^rd^ abdominal segments using a 10 microliter syringe and a disposable sterile 30 G needle. For wounding alone, a disposable sterile 30 G needle was inserted between the 2^nd^ and 3^rd^ abdominal segments, but no material was delivered.

### RNA Isolation, reverse-transcription and quantitative PCR for Gene Expression Analysis

Dissected material was placed into RNAlater (Invitrogen, San Diego, CA) for storage and RNA was prepared from bees by manually crushing the tissue of interest with a disposable pestle in Trizol Reagent (Invitrogen, San Diego, CA) and extracting the RNA as per the manufacturer’s instructions. RNA was subsequently DNAseI treated by RQ1 RNase-Free DNase (Promega, Madison, WI) and quantified. cDNA was synthesized using approximately 1 μg of RNA with the iScript cDNA Synthesis Kit (Biorad, Hercules, CA). Typically, 1 μl of cDNA was then used as a template for quantitative PCR to determine the levels of expression of genes of interest using the iQ SYBR Green Supermix (Biorad, Hercules, CA) in an iCycler thermo-cycler (Biorad, Hercules, CA). Primer sequences used in this study are in Supplemental Table 1. When possible, primers amplifying regions spanning introns were used. The difference between the threshold cycle number for *β-actin* and that of the gene of interest was used to calculate the level of that gene relative to *β-actin* using the ΔΔC_T_ method.

### Xbp1 mRNA Splicing

cDNA from above was used as template for a PCR using primers (Forward, 5′-ctgtgctgtgtcctcactgg-3′ and Reverse 5′- tcaagaggaagtagatggtcagaa-3′) that spanned the predicted splice sites. PCR products were run on a 2.5% Agarose gel to separate spliced from unspliced *Xbp1*.

### Statistical Analysis

All gene expression data were generated by processing and analyzing individual bees (where n denotes the number of bees in each treatment group) and then pooling data for a given experiment. Graphs show representative results from one of multiple trials. For analysis, data was log10 transformed and compared using unpaired t-tests with Welch’s correction as values fit normal distributions. Normality was assessed using Shapiro-Wilk tests. When more than two groups were being compared, data was compared using one way ANOVA with Tukey’s multiple comparison test.

## Electronic supplementary material

Supplementary Info
