# Supplementary material for: The heat shock response and humoral immune response are mutually antagonistic in honey bees
Source: Sci Rep. 2017 Aug 18;7:8850. doi: 10.1038/s41598-017-09159-4 (PMC5562734; doi:10.1038/s41598-017-09159-4)
Supplement: Supplementary file 1 — Supplementary Info [file 41598_2017_9159_MOESM1_ESM.pdf]

# The heat shock response and humoral immune response are mutually antagonistic in honey bees

Mia McKinstry[1], Charlie Chung[2], Henry Truong[1], Brittany A. Johnston[3], and Jonathan W. Snow[1]*

## Supplemental Information

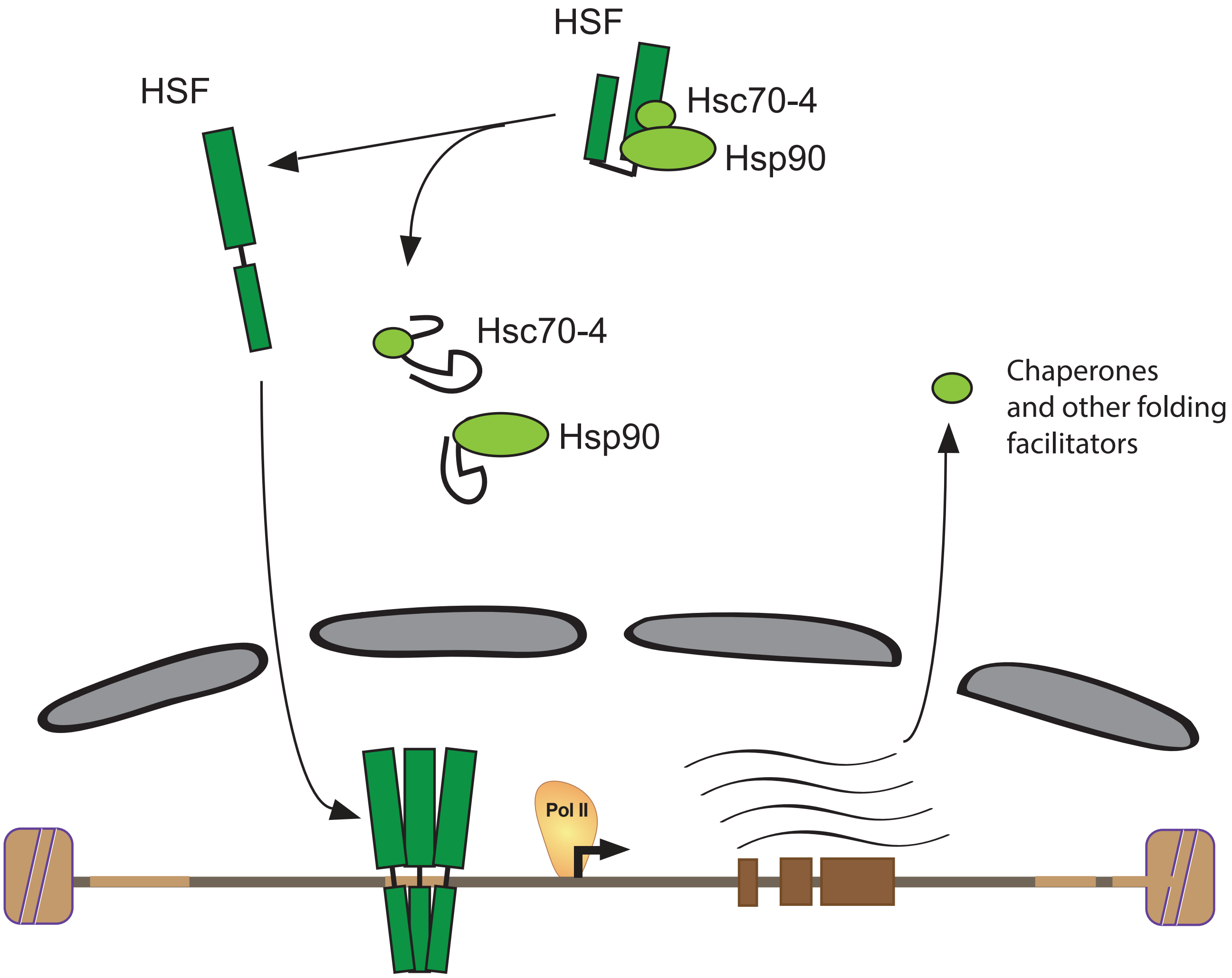


**Supplemental Figure 1.** Diagram of components of the HSR pathway conserved in honey bees.

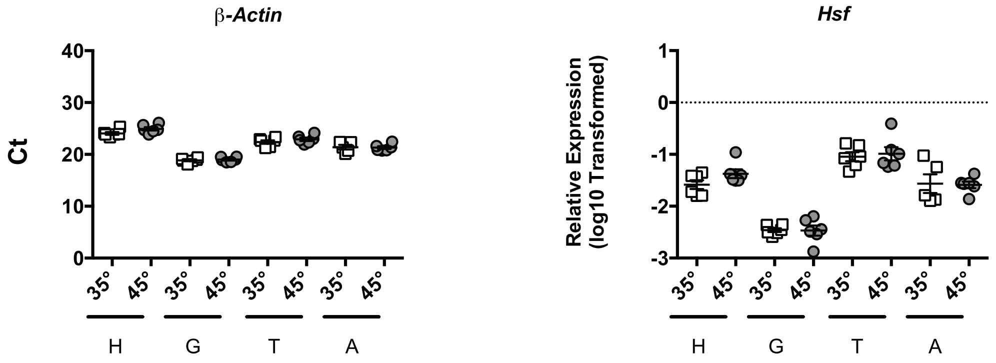


**Supplemental Figure 2.** Mean threshold cycle (Ct) for *β-actin* and transcript levels of HSR transcriptional regulator *Hsf* relative to *β-actin* in head tissue (H, predominantly brain and sensory organ tissue), midgut (G), thorax tissue (T, predominantly flight muscle), and abdominal wall (A, predominantly fat body) from bees maintained at 35° C or heat-shocked at 45°. Symbols represent expression values of the genes of interest calculated using the ΔΔCT method for individual bees. Mean ± SEM is also shown. Statistical significance was assessed using unpaired t-tests with Welch's and is noted as *$p < 0.05$, and **$p < 0.01$.

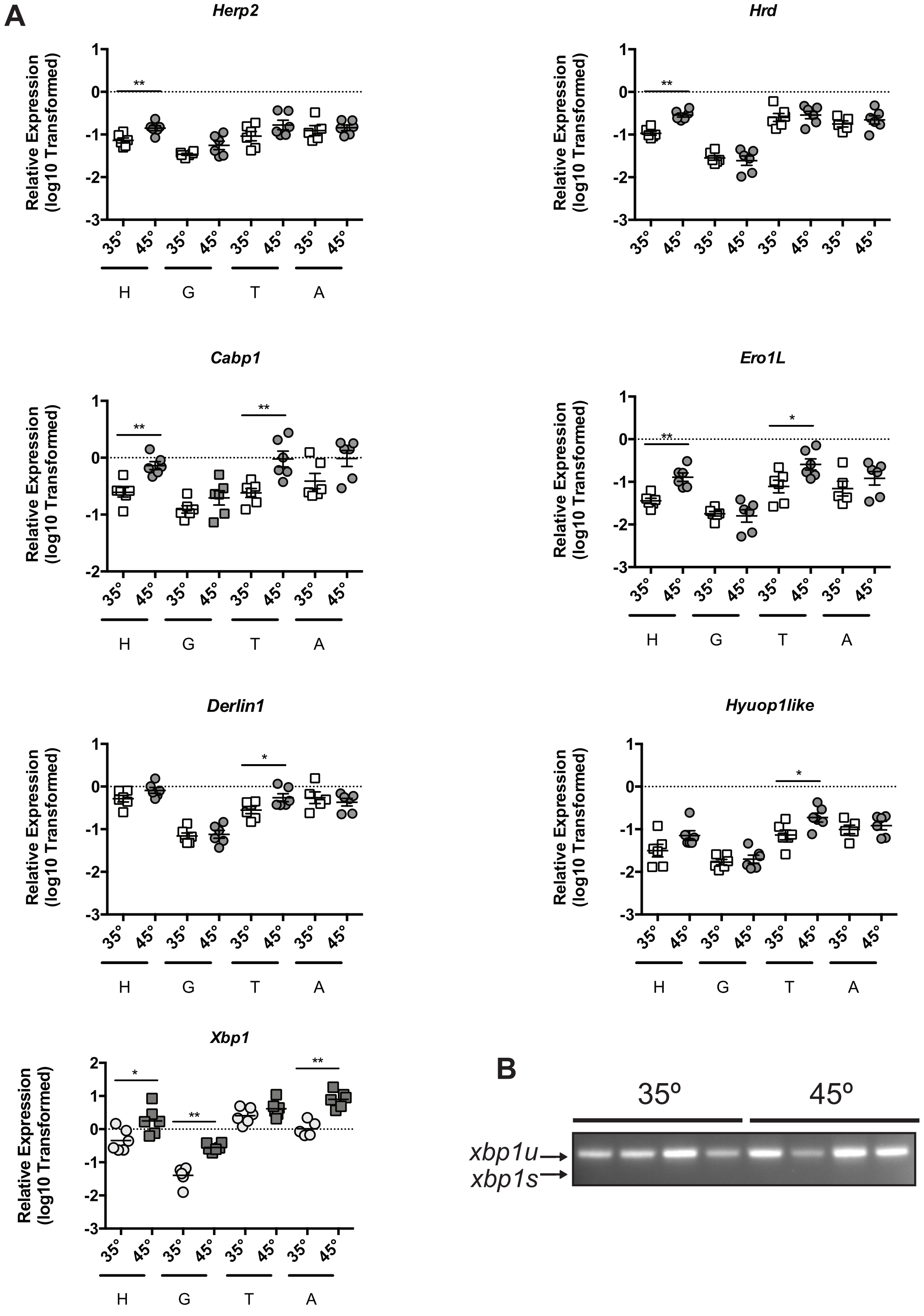


**Supplemental Figure 3.** (A) Transcript levels of UPRER target genes *Herp2*, *Hrd*, *Cabp1*, and *Ero1L*, *Derlin1*, *Hyoup1* and transcriptional regulator *Xbp1* relative to *β-actin* in head tissue (H, predominantly brain and sensory organ tissue), midgut (G), thorax tissue (T, predominantly flight muscle), and abdominal wall (A, predominantly fat body) from bees maintained for four hours in cages at either 35 ° or 45 °C . Symbols represent expression values of the genes of interest calculated using the ΔΔCT method for individual bees. Mean ± SEM is also shown. Statistical significance was assessed using unpaired t-tests with Welch's and is noted as $*p < 0.05$, and $**p < 0.01$. (B) *Xbp1* mRNA splicing by IRE1 in midgut tissue from individual bees maintained for 4 hours at 35° or 45° C.

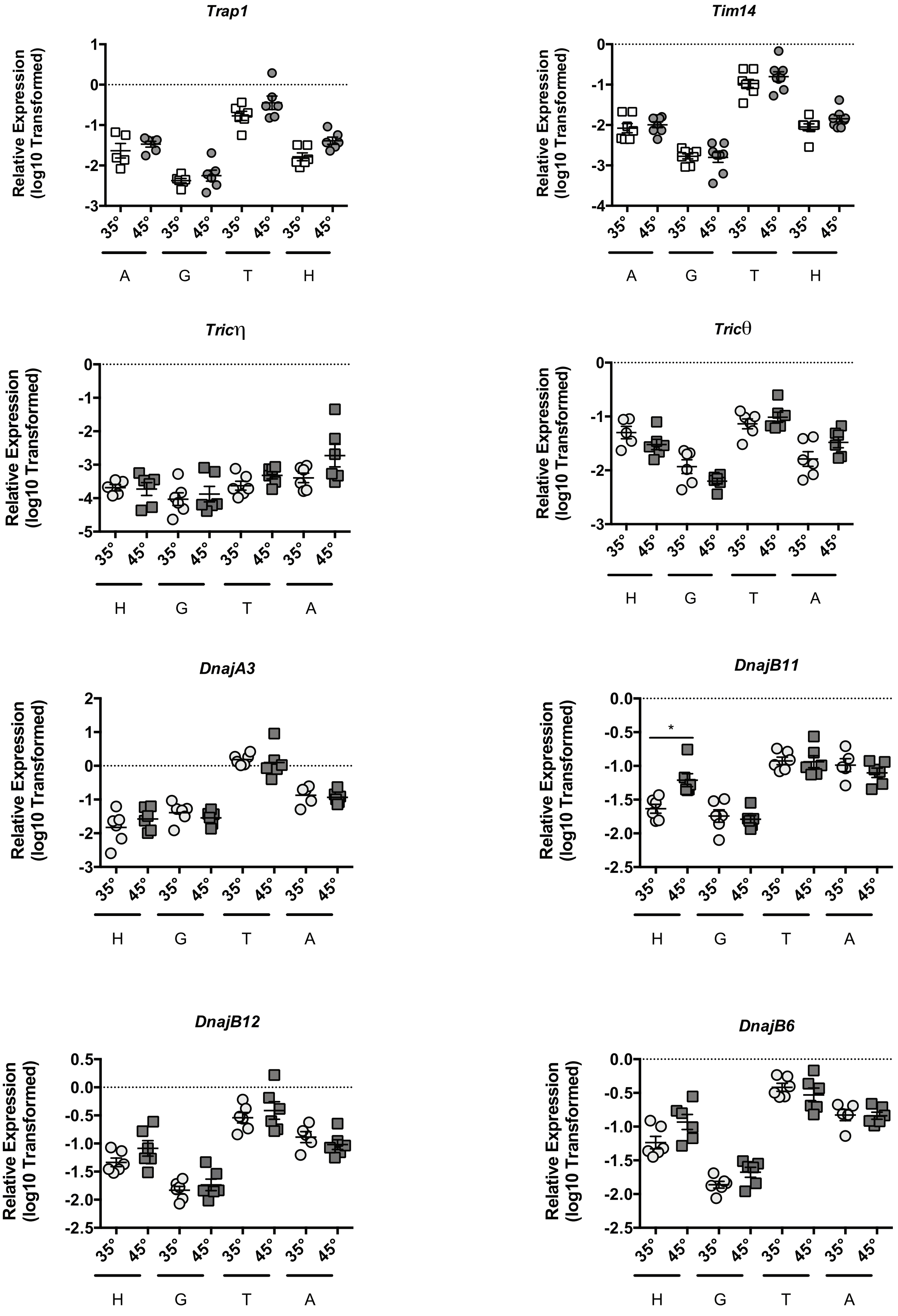


**Supplemental Figure 4.** Transcript levels of UPRMT target genes *Trap1*, *Tim14*, the TRiC genes *Tricη* and *Tricθ*, and the DNAJ-containing proteins *DnajA3*, *DnajB11*, *DnajB12*, *DnajB6* relative to *β-actin* in head tissue (H, predominantly brain and sensory organ tissue), midgut (G), thorax tissue (T, predominantly flight muscle), and abdominal wall (A, predominantly fat body) from bees maintained for four hours in cages at either 35 ° or 45 °C . Symbols represent expression values of the genes of i nterest calculated using the ΔΔCT method for individual bees. Mean ± SEM is also shown. Statistical significance was assessed using unpaired t-tests with Welch's and is noted as $*p < 0.05$, and $**p < 0.01$.

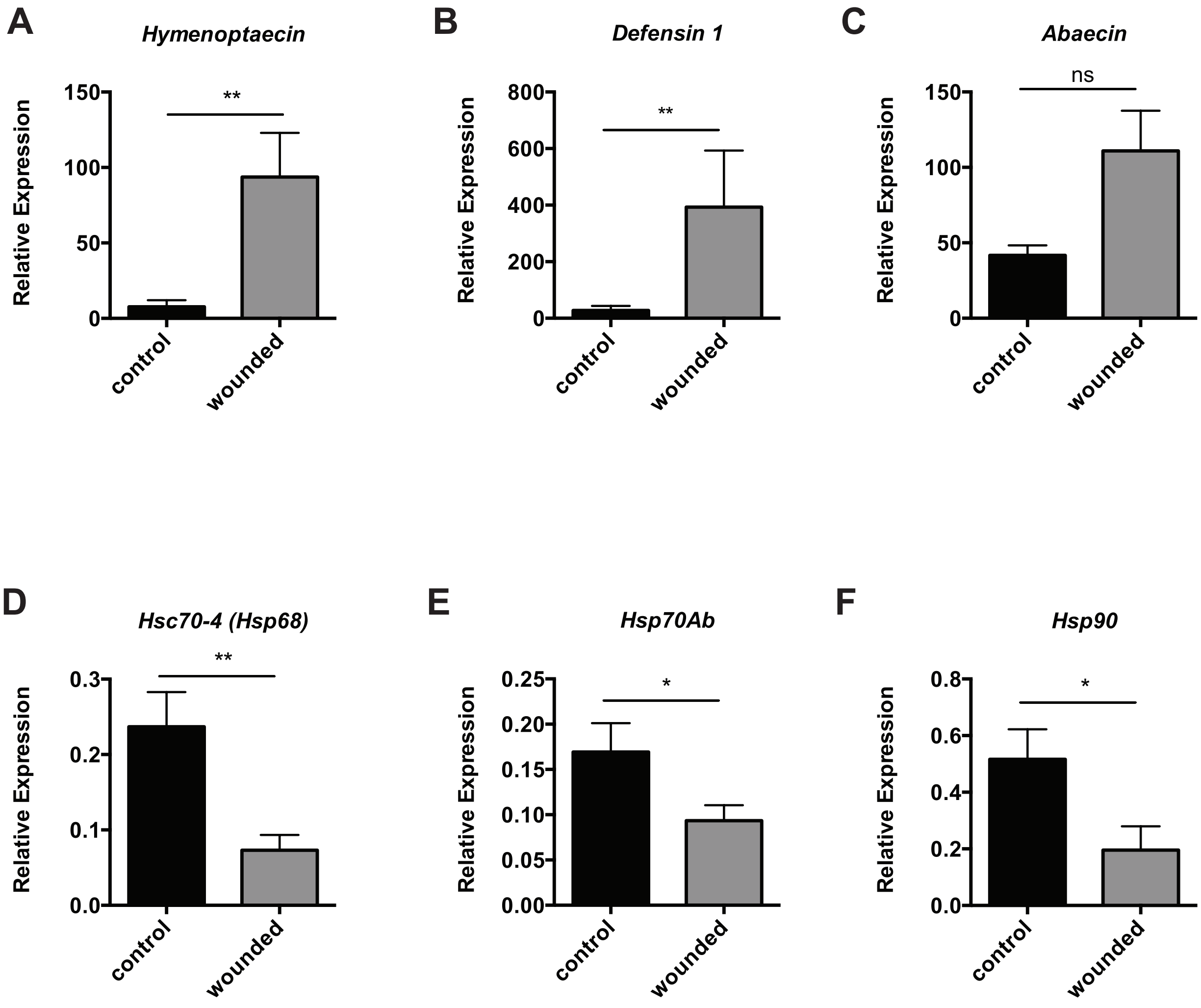


**Supplemental Figure 5.** Wounding represses select HSR target genes at the site of injury. Transcript levels of *Hymenoptaecin* (A), *Defensin 1* (B), and *Abaecin* (C), *Hsc70-4* (D), *Hsp70Ab* (E), and *Hsp90* (F) relative to *β-actin* in abdominal wall (predominantly fat body) from bees receiving a sterile wound or control bees after four hours. Bar and error bars represent the Mean ± SEM for expression values of the genes of interest calculated using the ΔΔCT method. Statistical significance was assessed using unpaired t-tests with Welch’s and is noted as $*p < 0.05$, and $**p < 0.01$.

**Supplemental Table 1. Primer Sequences Developed for Use in this Study**

| Name | Gene ID | Role | 5' primer | 3' primer | Reference |
|---|---|---|---|---|---|
| *Heat shock factor (Hsf)* | 411854 | signal transduction / transcription factor | AAGAAACGCGGATGTAAGGGT | GCCATTGCTGCGAGTTTTGA | this study |
| *Heat shock protein 90 (Hsp90)* | 408928 | chaperone | CATGGCTAATGCCGGAGAGG | CTGCACCAGCTTGAAGAGC | this study |
| *Heat shock protein 83-like (Hsp83)* | 411700 | chaperone | TACTCAACGCGCGTCTTCAT | TGAGCAATCTCTGCCTGGAA | this study |
| *Heat shock protein 70Ab-like (Hsp70Ab-like)* | 410620 | chaperone | CTTGCGTGGGTGTTTTCCAG | TATCATCGAAGCGACGACCG | this study |
| *Heat shock protein 70Cb ortholog (Hsp110)* | 408706 | chaperone | CATGGCTGCTATGTCCGTGA | TTTTTGGCTGCCACACCAAG | this study |
| *Heat shock protein cognate 4 (Hsc70-4, Hsp68)* | 409418 | chaperone | CTTGCGTGGGTGTTTTCCAG | TATCATCGAAGCGACGACCG | Johnston, 2016 |
| *Heat shock protein 10 (Hsp10)* | 552531 | chaperone | ACGCCATGCATGATCTTAAAGC | TGCCAGTATATCTGACTCACGG | this study |
| *DnaJ protein homolog 1-like (DnajB4)* | 411071 | chaperone | CACAGCTTACGCGACACTTT | TCTCTTCTGCTCCAGCACTTC | this study |
| *DnaJ homolog subfamily A member 1 (DnajA1)* | 724368 | chaperone | AGGCTCGTATTTCTGCACTCA | AGGTACGTGCAGCTTCATCC | this study |
| *Heat shock 70 kDa protein cognate 3* | 409587 | chaperone | TCGAAGTCGTGCTGAGAGTA | CCATCAGCAGTAAATGCCACA | Johnston, 2016 |
| *Glycoprotein 93 (Gp93)* | 412150 | chaperone | TAGCCGCGAAGGATCTCGAA | CGATTGACTTCGGTTTGAAAAGT | Johnston, 2016 |
| *p58ipk* | 413441 | chaperone | TCAGGAACCAACTCAGGCAA | GATGCTGTTCTTGGCACACC | Johnston, 2016 |
| *T-complex chaperonin Epsilon* | 409825 | chaperonin subunit | TGCCCATTTGAACCTCCCAA | GGAACAATACGTCCACCCGT | this study |
| *T-complex chaperonin Delta* | 551275 | chaperonin subunit | GATGGGCGAAATGCTCATGG | CGTTCTGCAGCTTCCAAAAGA | this study |
| *T-complex chaperonin Eta* | 550694 | chaperonin subunit | ACTTCCGTTAAGTGTGAACGAT | TTGTGCAGCATCTGTGCCTT | this study |
| *T-complex chaperonin Theta* | 551436 | chaperonin subunit | CGATGTCGCTGTAATCGCAA | GCCATTTGGGCCATAAGCAG | this study |
| *Heat shock protein cognate 5 (Hsc70-5)* | 408605 | chaperone | CAAGATGCTGACTGCTGCAAG | TGGAGTCGTTCGTGATCCTTC | this study |
| *Heat shock protein 60 (Hsp60)* | 409384 | chaperone | TACTAGCGAGACAACGTGCG | TTGTAATTGGCGCAAGGCTG | this study |
| *Hypoxia up-regulated protein 1-like (Hyuop1-like)* | 551763 | chaperone | ACGTACGTGTACCTATGCCT | CCCACTACTTGAGCATCTTCTCC | this study |
| *Tumor Necrosis Factor Receptor-Associated Protein-1 (Trap)* | 550968 | chaperone | CGAAAGCATGGCTACCGTTC | TTCAAGTGCATCACTCGCATT | this study |
| *Protein tumorous imaginal discs, mitochondria (DnajA3)* | 411359 | chaperone | CGGCCTGGTACTTCATCTCA | CGCTTGAGTTTTTACCCTTGGA | this study |
| *DnaJ homolog subfamily B member 11* | 552223 | chaperone | AACGTCTGCTAGAATGGCAGC | ATTGCATGTTGGCTTGCTGTT | this study |
| *DnaJ homolog subfamily B member 12* | 413120 | chaperone | ATGAGGCGGAGCGATGTATG | TGGATGCAGTTTGACGTTTCC | this study |
| *DnaJ homolog subfamily B member 6-like/6-B* | 408966 | chaperone | AGCACGTCCTTCTTTGGTCC | CGAGATATCGCTGGCACTGT | this study |
| *Mitochondrial import inner membrane translocase (Tim14)* | 726653 | chaperone | TGAAGATAACGCGTGCCAGT | ACTAGCTTCACGTCGTGTCA | this study |
| *Abaecin* | 406144 | antimicrobial peptide | CGTATGTACCATTACCAAATGTACCA | GACCAGGAAACGTTGGAAAC | this study |
| *Defensin 1* | 406143 | antimicrobial peptide | TGCGCTGCTAACTGTCTCAG | AATGGCACTTAACCGAAACG | Evans, 2006 |
| *Hymenoptaecin* | 406142 | antimicrobial peptide | CTCTTCTGTGCCGTTGCATA | GCGTCTCCTGTCATTCCATT | Evans, 2006 |
| *Universal Bacteria 16S* | NA | bacteria | ACTCCTACGGGAGGCAGCAGT | ATTACCGCGGCTGCTGGC | Hartman, 2009 |
| *Gfp* | NA | db11 bacteria gfp | GTCAGTGGAGAGGGTGAAGG | TACATAACCTTCGGGCATGG | this study |

| Color | Legend |
|---|---|
| Green | Signal Transduction / Transcription Factor |
| Blue | Predicted Core HSR Target Genes |
| Pink | Other Potential HSR Target Genes |
| Tan | Other Genes in This Study |

**Supplemental Table 2.** All genes encoding HSP70 proteins in the honey bee genome.

| Name | *A. mellifera* Gene ID | Gene of H. sapiens closest homolog | Signal Sequence (SignalP 4.1) | M, S (TargetP 1.1) | Prosite (KDEL) | localization | Heat Shock |
|---|---|---|---|---|---|---|---|
| *Heat shock protein 70Ab-like (Hsp70Ab-like)* | 410620 | *Hspa1a, Hspa8, Hspa1* | N | N | N | C | increased |
| *Heat shock protein 70Cb ortholog (Hsp110)* | 408706 | *Hsph1* | N | N | N | C | increased |
| *Heat shock protein cognate 4 (Hsc70-4, Hsp68)* | 409418 | *Hspa1a, Hspa8, Hspa1* | N | N | N | C | increased |
| *Heat shock 70 kDa protein cognate 3 (Hsc70-3)* | 409587 | *Grp78* | Y | S | Y | ER | increased |
| *Heat shock protein cognate 5 (Hsc70-5)* | 408605 | *mHsp70* | N | M | N | M | increased |
| *Hypoxia up-regulated protein 1-like (Hyuop1-like)* | 551763 | *Grp170* | Y | N | N | ER | no change |

C = cytoplasm
ER = endoplasmic Reticulum
M = mitochondria
S = secretory
Y = found
N = none found
NT = not tested

| **Supplemental Table 3.** All genes encoding HSP90 proteins in the honey bee genome. | | | | | | | |
|---|---|---|---|---|---|---|---|
| **Name** | ***A. mellifera* Gene ID** | **Gene of H. sapiens closest homolog** | **Signal Sequence (SignalP 4.1)** | **M, S (TargetP 1.1)** | **Prosite (KDEL)** | **localization** | **Heat Shock** |
| *Heat shock protein 90 (Hsp90)* | 408928 | *Hsp90* | N | N | N | C | increased |
| *Heat shock protein 83-like (Hsp83like)* | 411700 | *Hsp90* | N | N | N | C | no change |
| *Glycoprotein 93 (Gp93)* | 412150 | *Grp94* | Y | S | Y | ER | increased |
| *Tumor Necrosis Factor Receptor-Associated Protein-1 (Trap1)* | 550968 | *Trap1* | N | M | N | M | no change |

C = cytoplasm
ER = endoplasmic Reticulum
M = mitochondria
S = secretory
Y = found
N = none found
NT = not tested

**Supplemental Table 4.** All genes encoding DNAJ-containing proteins in the honey bee genome.

| Name | *A. mellifera* Gene ID | class | client binding | Gene of H. sapiens closest homolog | Signal Sequence (SignalP 4.1) | M, S (TargetP 1.1) | Prosite (KDEL) | localization | Heat Shock |
|---|---|---|---|---|---|---|---|---|---|
| *DnaJ homolog subfamily A member 1* | 724368 | A | promiscuous | *DnajA1* | N | N | N | C | increased |
| *protein tumorous imaginal Discs, mitochondiria* | 411359 | A | promiscuous | *DnajA3* | N | M | N | M/ER | no change |
| *DnaJ protein homolog 1-like* | 411071 | B | promiscuous | *DnajB4* | N | N | N | C | no change |
| *DnaJ homolog subfamily B member 11* | 552223 | B | promiscuous | *DnajB11* | N | S | N | ER | no change |
| *DnaJ homolog subfamily B member 12* | 413120 | B | wide | *DnajB12* | N | N | N | C/Era | no change |
| *DnaJ homolog subfamily B member 6-like/6-B* | 408966 | B | wide | *DnajB2 (Hsj1)* | N | N | N | C/ER | no change |
| *DnaJ homolog Dnj-5* | 409594 | C | specific | *DnajC14* | N | N | N | C/ER/M | NT |
| *DnaJ homolog subfamily C member 16* | 412118 | C | specific | *DnajC16* | N | N | N | U | NT |
| *DnaJ homolog subfamily C member 17* | 552846 | C | specific | *DnajC17* | N | N | N | U | NT |
| *DnaJ homolog subfamily C member 21* | 100578360 | C | specific | *DnajC21* | N | N | N | U | NT |
| *DnaJ homolog subfamily C member 3* | 413441 | C | specific | *DnajC3 (p58iik)* | Y | S | N | ER | increased |
| *DnaJ homolog subfamily C member 5* | 724965 | C | specific | *DnajC5* | N | N | N | exo | NT |
| *DnaJ homolog subfamily C member 7* | 410037 | C | specific | *DnajC7* | N | N | N | C | NT |
| *Cyclin-G-associateD kinase* | 413462 | C | specific | *DnajC26* | N | N | N | C/N | NT |
| *DnaJ homolog subfamily C member 22* | 410730 | C | specific | *DnajC22* | N | N | N | C | NT |
| *DnaJ homolog subfamily C member 24* | 724329 | C | specific | *DnajC24* | N | N | N | C | NT |
| *DnaJ homolog subfamily B member 13* | 727642 | C | unclear | *DnajB13* | N | N | N | C | NT |
| *DnaJ homolog subfamily C member 11* | 409991 | C | unclear | *DnajC11* | N | N | N | M | NT |
| *DnaJ homolog subfamily C member 13* | 411060 | C | unclear | *DnajC13 (Rem8)* | N | N | N | C/M | NT |
| *DnaJ homolog subfamily C member 8* | 410294 | C | unclear | *DnajC8 (Sfp3b)* | N | N | N | C | NT |
| *DnaJ homolog subfamily C member 9-like* | 409892 | C | unclear | *DnajC9* | N | N | N | N | NT |
| *DnaJ homolog subfamily C member 1-like* | 552151 | C | no | *DnajC1 (Erdj1)* | Y | S | N | ER | NT |
| *J Domain-containing protein* | 408863 | C | no | *DnajC12 (Jpd1)* | N | N | N | U | NT |
| *Mitochondrial import inner membrane translocase* | 726653 | C | no | *DnajC19 (Tim14)* | N | S | N | M | no change |
| *DnaJ homolog subfamily C member 2* | 413209 | C | no | *DnajC2 (Zuotin)* | N | N | N | C | NT |
| *SEC63 homolog* | 412505 | C | no | *DnajC23 (Sec63)* | N | N | N | U | NT |
| *DnaJ homolog subfamily C member 18* | 102654159 | ? | ? | *ND* | N | N | N | U | NT |

C = cytoplasm
ER = endoplasmic Reticulum
M = mitochondria
S = secretory
Y = found
N = none found
NT = not tested

**Supplemental Table 5.** All genes encoding alpha-crystallin domain containing proteins in the honey bee genome.

| **Name** | ***A. mellifera* Gene ID** | **Gene of H. sapiens closest homolog** | **Signal Sequence (SignalP 4.1)** | **M, S (TargetP 1.1)** | **Prosite (KDEL)** | **localization** | **Heat Shock** |
|---|---|---|---|---|---|---|---|
| *Heat shock protein beta-1* | 408875 | *alpha-crystallin B chain* | N | N | N | C | NT |
| *Very-long-chain (3R)-3-hydroxyacyl-CoA dehydratase* | 413078 | *Very-long-chain (3R)-3-hydroxyacyl-CoA dehydratase* | N | N | N | C | NT |
| *SGT1 homolog* | 551201 | *SGT1* | N | N | N | C | NT |
| *Lethal(2)essential for life-like (l(2)efl)* | 724488 | *HSPB1, clust* | N | N | N | C | NT |
| *Lethal(2)essential for life-like (l(2)efl)* | 724405 | *HSPB5 (CRYAB) clust* | N | N | N | C | NT |
| *Lethal(2)essential for life-like (l(2)efl)* | 724367 | *HSPB5 (CRYAB) clust* | Y | S | N | ER | NT |
| *Lethal(2)essential for life-like (l(2)efl)* | 724274 | *HSPB5 (CRYAB) clust* | N | N | N | C | NT |
| *Lethal(2)essential for life-like (l(2)efl)* | 410857 | *HSPB5* | N | N | N | C | NT |
| *Lethal(2)essential for life-like (l(2)efl)* | 724449 | *HSPB1 clust* | N | N | N | C | NT |
| *Lethal(2)essential for life-like (l(2)efl)* | 412197 | *HSPB5 (CRYAB)* | N | N | N | C | NT |
| *uncharacterized LOC410087* | 410087 | *HSPB5 (CRYAB)* | N | M | N | C | NT |
| *PIH1 domain containing 1* | 726775 | *PIH1 domain containing 1* | N | N | N | C | NT |
| *PIH1D3-like* | 102655121 | *PIH1D3* | N | N | N | C | NT |

C = cytoplasm
ER = endoplasmic Reticulum
M = mitochondria
S = secretory
Y = found
N = none found
NT = not tested

**Supplemental Table 6.** All genes encodingchaperonin proteins in the honey bee genome.

| Name | *A. mellifera* Gene ID | *H. sapiens* closest homolog | Signal Sequence (SignalP 4.1) | M, S (TargetP 1.1) | Prosite (KDEL) | localization | Heat Shock |
|---|---|---|---|---|---|---|---|
| *T-complex protein 1 subunit alpha* | 409134 | *TCP1 alpha* | N | M | N | C/M | NT |
| *T-complex protein 1 subunit beta* | 409809 | *TCP1 beta* | N | N | N | C | NT |
| *T-complex chaperonin delta* | 551275 | *TCP1 delta* | N | N | N | C | up |
| *T-complex chaperonin epsilon (Cct5)* | 409825 | *TCP1 epsilon* | N | N | N | C | up |
| *T-complex protein 1 subunit gamma* | 409296 | *TCP1 gamma* | N | N | N | C | NT |
| *T-complex protein 1 subunit eta* | 550694 | *TCP1 eta* | N | N | N | C | no change |
| *T-complex protein 1 subunit theta* | 551436 | *TCP1 theta* | N | N | N | C | no change |
| *T-complex protein 1 subunit zeta* | 551295 | *TCP1 zeta* | N | N | N | C | NT |
| *Heat shock protein 60 (Hsp60)* | 409384 | *Hsp60* | N | M | N | M | up |
| *Heat shock protein 10 (Hsp10)* | 552531 | *Hsp10* | N | M | N | M | up |

C = cytoplasm
ER = endoplasmic Reticulum
M = mitochondria
S = secretory
Y = found
N = none found
NT = not tested
